# Adenovirus-mediated Foxp3 expression in lung epithelial cells reduces airway inflammation in ovalbumin and cockroach-induced asthma model

**DOI:** 10.1038/emm.2016.83

**Published:** 2016-09-16

**Authors:** Soojin Park, Hwan-Suck Chung, Dasom Shin, Kyung-Hwa Jung, Hyunil Lee, Junghee Moon, Hyunsu Bae

**Affiliations:** 1 Department of Science in Korean Medicine, Kyung Hee University, Dongdaemoon-gu, Seoul, Republic of Korea; 2 Korean Medicine (KM)-Application Center, Korea Institute of Oriental Medicine (KIOM), Daegu, Republic of Korea; 3 Department of Biological Sciences in Korean Medicine, Kyung Hee University, Seoul, Republic of Korea

## Abstract

Foxp3 is a master regulator of CD4^+^CD25^+^ regulatory T-cell (Treg) function and is also a suppressor of SKP2 and HER2/ErbB2. There are an increasing number of reports describing the functions of Foxp3 in cell types other than Tregs. In this context, we evaluated the functions of Foxp3 in ovalbumin- and cockroach-induced asthma models. Foxp3-EGFP-expressing adenovirus or EGFP control adenovirus was administered intratracheally (i.t.), followed by challenge with ovalbumin (OVA) or cockroach extract to induce asthma. Th2 cytokine and immune cell profiles of bronchoalveolar lavage fluid (BALF), as well as serum IgE levels, were analyzed. Histological analyses were also conducted to demonstrate the effects of Foxp3 expression on airway remodeling, goblet cell hyperplasia and inflammatory responses in the lung. Adenoviral Foxp3 was expressed only in lung epithelial cells, and not in CD4^+^ or CD8^+^ cells. BALF from Foxp3 gene-delivered mice showed significantly reduced numbers of total immune cells, eosinophils, neutrophils, macrophages and lymphocytes in response to cockroach allergen or OVA. In addition, Foxp3 expression in the lung reduced the levels of Th2 cytokines and IgE in BALF and serum, respectively. Moreover, histopathological analysis also showed that Foxp3 expression substantially inhibited eosinophil infiltration into the airways, goblet cell hyperplasia and smooth muscle cell hypertrophy. Furthermore, when Tregs were depleted by diphtheria toxin in Foxp3^DTR^ mice, the anti-asthmatic functions of Foxp3 were not altered in OVA-challenged asthma models. In this study, our results suggest that Foxp3 expression in lung epithelial cells, and not in Tregs, inhibited OVA- and cockroach extract-induced asthma.

## Introduction

Mutation of the transcription factor Forkhead box P3 (Foxp3) leads to fatal autoimmune diseases in mice and humans.^1, 2^ Although Foxp3 was initially shown to be a key transcription factor in the control of regulatory T-cell (Treg) function,^3^ there are an increasing number of reports describing the functions of Foxp3 in other cell types. Specifically, Foxp3 is known to be expressed in epithelial cells of many lineages, including breast.^4^ In addition, by using *Rag2*^*−/−*^ mice, Chen *et al.*,^4^ also showed that Foxp3 is expressed in the airway epithelium. Moreover, it has also been demonstrated that Foxp3 expression represses the epidermal growth factor receptor (HER2/ErbB2) and SKP2 oncogenes in breast cancer cells.^5, 6^ Furthermore, we have previously studied the expression and functions of Foxp3 in microglia.^7, 8^ In this context, in the present study, we examine the function of the Foxp3 gene in asthma models.

Asthma is a chronic airway inflammatory disease characterized by increased mucus production, intermittent airway obstruction and airway hyperresponsiveness.^9, 10^ Airway remodeling can also occur and is characterized by structural and morphologic changes to the airways, including subepithelial fibrosis, epithelial hypertrophy, goblet cell hyperplasia and smooth muscle cell hypertrophy.^11, 12^

The airway epithelium is a barrier to potentially toxic or damaging environmental insults. In moderate and severe childhood asthma, however, epithelial injury and aberrant repair are commonly observed, and there is much evidence to suggest that the barrier function of the airway epithelium is impaired in this disease.^13^ Moreover, airway epithelial cells from mouse models of house dust mite-induced allergic asthma secrete CC chemokine ligand 2 (CCL2) and CCL20, which recruit monocytes and immature dendritic cells (DCs) to the lung.^14, 15^ The airway epithelium also releases various cytokines such as IL-33, IL-1β and thymic stromal lymphopoietin to activate DCs to promote Th2 immune responses.^14, 16^ Thus, there is evidence to suggest that asthma is initiated following epithelial cell injury.

Adenoviruses are one of the most commonly used vectors for gene therapy. Adenoviral vectors transmit their genes to the host nucleus, but the genetic material is not integrated into the host chromosome. Therefore, adenovirus-delivered genes do not perturb vital cellular genes or processes.^17^ Furthermore, adenoviruses can also transduce genes to non-dividing cells. In this study, we explored the therapeutic potential of adenovirus-mediated Foxp3 gene delivery in an asthma model.

## Materials and methods

### Adenovirus construction

The AdEasy System (Agilent Technologies, La Jolla, CA, USA) was used to construct a recombinant adenoviral vector. Briefly, the mouse Foxp3 gene from the Foxp3 pcDNA plasmid^18^ was inserted into a recombinant adenoviral shuttle vector (pShuttle-IRES-GFP, Agilent Technologies), and co-transformed into *Escherichia coli* BJ5183 cells together with an adenoviral backbone plasmid (AdEasy). The recombinant plasmid was then transfected into the HEK-293 adenovirus packaging cell line, and viruses were purified from infected cells 48 h after infection using a virus purification kit (Virapur, San Diego, CA, USA). The purified virus was stored at−80 °C until further use. Viral titers were measured using a standard end-point dilution assay with HEK-293 cells.

### Mice

Female C57BL/6 and Balb/c mice (6–7 weeks of age) were purchased from Charles River Korea (OrientBio, Sungnam, Korea). Foxp3^*EGFP/DTR*^C57BL/6-Tg mice (Foxp3^*DTR/EGFP*^23.2Spar/Mmjax) were purchased from The Jackson Laboratory (Bar Harbor, ME, USA). All mice were kept under pathogen-free conditions with air conditioning and a 12-h light/dark cycle. All mice had *ad libitum* access to food and water during the experiments. The study was conducted according to the Rules for Animal Care and the Guiding Principles for Animal Experiments Using Animals by the University of Kyung Hee Animal Care and Use Committee and (KHUASP (SE)-11-025).

### Lung dissociation and flow cytometry to detect Foxp3-expressing adenovirus

Mice were infected once i.t. with the Foxp3-expressing adenovirus (Ad-Foxp3-EGFP, 5 × 10^8^ pfu). Control mice received the same dose of control virus (Ad-EGFP). To assess Ad-Foxp3-EGFP infection, mice were killed 3 days post infection. The lungs were excised and processed for EGFP expression by flow cytometry. The lungs were removed and washed with phosphate-buffered saline (PBS) to remove blood. A single-cell pneumonocyte suspension was prepared using a Lung Dissociation Kit (Miltenyi Biotec, Bergisch-Gladbach, Germany) according to the manufacturer's instructions. Single-cell pneumocyte suspensions obtained from C57/BL6 mice were labeled with APC-conjugated anti-CD326 (Ep-CAM) (BioLegend, San Diego, CA, USA), APC-conjugated anti-CD4 and PE-conjugated anti-CD8 monoclonal antibodies (both from eBioscience, San Diego, CA, USA) using standard staining methods. The percentage of cells staining positive with a particular reagent was analyzed with a FACS Calibur flow cytometer using CellQuest software (BD Biosciences, San Jose, CA, USA). The results were generated in graphical and tabular formats using FlowJo software (Tree Star Inc., Ashland, OR, USA).

### Confocal microscopy

To examine EGFP expression by confocal microscopy, mice were infected i.t. with Ad-Foxp3-EGFP or control adenovirus as described and were killed 3 days post infection. Lungs were excised, fixed overnight in 4% buffered paraformaldehyde at 4 °C, stored in a 30% sucrose solution at 4 °C until they settled to the bottom of their container, and frozen-sectioned on a sliding microtome into 30-μm-thick coronal sections. Lung tissue was washed with PBS, mounted with Vectashield mounting medium with DAPI (Vector Laboratories Inc., Burlingame, CA, USA) and analyzed by confocal microscopy (Zeiss LSM Pascal 5, Heidelberg, Germany).

### *In vivo* experimental design

For experiments concerning the animal model of cockroach allergen (CKA)-induced asthma, the study schedule was modified from the methods of McGee and Agrawal.^19^ Briefly, the mice were sensitized by intraperitoneal (i.p.) injection with 10 μg of CKA (Hollister-Stier, Spokane, WA, USA) in incomplete Freund's adjuvant (Sigma-Aldrich, St Louis, MO, USA) on days 0 and 14.^20^ Subsequently, mice received an intratracheal (i.t.) challenge with cockroach allergen (5% CKA in PBS) on days 22-31. The negative control mice were sensitized and challenged with PBS alone. For the animal model of ovalbumin (OVA)-induced asthma, mice were sensitized by i.p. injection of 0.1 mg of OVA (Sigma-Aldrich), together with 20 mg of aluminum hydroxide in 100 μl of PBS on days 0 and 14. Then, the mice were i.t. challenged with 1% OVA in 50 μl of PBS six times between days 22 and 31. For inhibition studies, both the negative control (CON) and CKA-exposed (CKA) groups were treated with only PBS by i.t. challenge, whereas the positive control (Dexa) group was treated with dexamethasone (10 mg kg^−1^ per day) suspended in PBS by oral gavage 1 day before challenge with 1% OVA or 5% CKA (day 21 or 31).

Twenty-four hours after the final challenge, mice were killed by i.p. injection of pentobarbital sodium (50 mg kg^−1^, Hanlim Pharm. Co., Seoul, Korea) and exsanguination without any previous intervention. The animal experiments were performed according to the guidelines recommended by Animal Research: Reporting *In Vivo* Experiments (ARRIVE). We performed each experiment twice.

### Administration of adenoviral vectors

Twenty-four hours before challenge with OVA or CKA (day 21 or 31), a micropipette was used to i.t. infect mice with Ad-Foxp3-EGFP (5 × 10^8^ pfu). Control mice received the same dose of control virus (Ad-EGFP).

### Bronchoalveolar lavage fluid

Bronchoalveolar lavage fluid (BALF) was collected by infusion and extraction of 1 ml of ice-cold PBS. This procedure was repeated three times, and lavages from individual mice were pooled. Recovered BALF (70–80%) was centrifuged at 1300 r.p.m. for 10 min. The cell pellets were then resuspended in 1 ml PBS and adhered to glass slides using cytocentrifugation. Total viable cell counts were determined using a hemocytometer with trypan blue exclusion. Differential counts of eosinophils, neutrophils, lymphocytes and macrophages were determined on Diff-Quick-stained (Life Technologies, Auckland, New Zealand) cytospin smears of BALF samples (5 × 10^5^/200 μl cells) from individual mice. BALF was then centrifuged and the supernatants were stored at −80 °C. The results are expressed as total cell number × 10^4^.

### Assessment of cytokine expression

The levels of IL-4, IL-5 and IL-13 in the BALF were assessed using a quantitative sandwich enzyme-linked immunoassay kit (BD Biosciences for IL-4, IL-5 and R&D Systems, Minneapolis, MN, USA for IL-13). A 96-well plate was coated overnight at 4 °C with anti-mouse IL-4, IL-5 or IL-13 monoclonal antibodies in coating buffer. After washing, the wells were blocked with 5% FBS in PBS and 1% BSA in PBS for 1 h at 4 °C and room temperature, respectively. Subsequently, wells were loaded with 100 μl of BALF and incubated for 2 h at room temperature. After washing, secondary peroxidase-labeled biotinylated anti-mouse IL-4, IL-5 or IL-13 monoclonal antibodies in their respective assay diluents were added for 1 h. Finally, the plates were treated with TMB substrate solution (KPL, San Diego, CA, USA) for 30 min, and the reaction was stopped by the addition of 50 μl TMB stop solution per well. The optical density was measured at 450 nm in a microplate reader (SOFT max PRO, version 3.1. software, CA, USA). All of the results were normalized to the total amount of BALF protein in each sample.

### Determination of IgE titers using ELISA

The total levels of IgE in serum were determined by ELISA. Blood was collected from the retro-orbital plexus of mice while under ether anesthesia. Serum samples were obtained by centrifugation and stored at −20 °C until use. The ELISA plates were coated overnight at 4 °C with anti-mouse IgE antibody (BD Pharmingen). The serum samples were then diluted 1:300 with 5% FBS in PBS (assay diluent), and IgE levels were measured using a standardized sandwich ELISA according to the manufacturer's protocol. The lower limit of detection for this ELISA was 1.5 ng ml^−1^. The optical density was measured at 450 nm using a microplate reader (SOFT max PRO, version 3.1. software).

### Histological examination

The lungs were removed, fixed in 4% paraformaldehyde, dehydrated and embedded in paraffin. The tissue was cut into 4-μm-thick sections and stained with hematoxylin and eosin (H&E) for evaluation of inflammation and periodic acid-Schiff (PAS) reagent for evaluation of goblet cells. The number of goblet cells within the bronchial epithelium was quantified as the percentage of PAS-positive cells. Four bronchioles randomly selected from each section of mouse lung tissue were used for the analysis, and the mean goblet cell number from each section was calculated.^21^ The diameters of bronchi and bronchioles exhibiting goblet cell metaplasia were determined using an Olympus BX51 microscope (Olympus, Tokyo, Japan) equipped with a DP71 digital camera (Olympus). Inflammation in H&E-stained lung sections was evaluated with a subjective score ranging from 0 to 5 by five independent, blinded readers. All sections were scored from 0 to 5 according to the following criteria: 0=normal; 1=very mild; 2=mild; 3=moderate; 4=marked; 5=severe inflammation.^22, 23^ For immunohistochemistry of myosin regulatory light polypeptide 9 (MYL9), the lung sections were treated with 0.3% H_2_O_2_ in methanol for 20 min to block endogenous peroxidases. The sections were subsequently incubated with anti-mouse MYL9 primary antibodies (1:50 dilution; Santa Cruz Biotechnology, Santa Cruz, CA, USA), biotinylated anti-goat IgG secondary antibodies, and avidin-peroxidase complex (Vectastatin ABC kit; Vector Laboratories, Burlingame, CA, USA). The slides were developed with the peroxidase substrate, 3′3-diaminobenzidine tetrachloride (DAB; Zymed Laboratories, South San Francisco, CA, USA). After immunohistochemical staining, the slides were counterstained with hematoxylin for 1 min and mounted with Canada balsam (Show Chemical Co. Ltd., Tokyo, Japan). Four bronchioles were randomly selected from each slide, and cross-sections of lung parenchyma were captured, digitized and evaluated using Image Pro-Plus 6.1 software (Media Cybernetics, Inc., Silver Spring, MD, USA).

### Statistical analysis

Statistical analyses were conducted using Prism 5 software (Graph Pad Software Inc., CA, USA). All values are presented as the means±s.e.m. The differences between the means of the control and treatment samples were determined by one-way ANOVA with Tukey's Multiple Comparison test. Statistical significance was defined as *P*<0.05.

## Results

### Ad Foxp3-EGFP or Ad-EGFP are only expressed in lung epithelial cells

**Figure 1:**
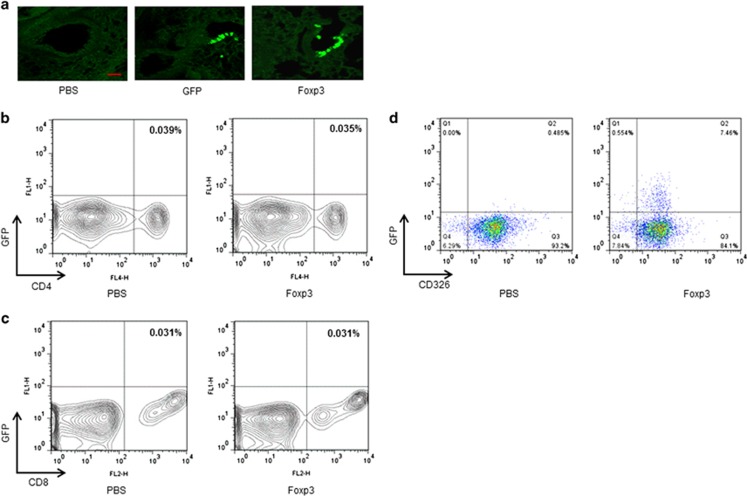
EGFP expression in epithelial cells. Fluorescence microscopy of epithelial cells 72 h after infection with Ad Foxp3-EGFP or Ad-EGFP. EGFP fluorescence is shown in green (**a**). Flow cytometry profiles of dissociated lung cells stained with anti-CD326 (Ep-CAM)-APC (**d**), anti-CD4-APC (**b**) and anti-CD8-PE monoclonal antibodies (**c**).

To investigate which cell types were infected following intratracheal Ad Foxp3-EGFP or Ad-EGFP treatment, we analyzed the EGFP expression in lung tissue by confocal microscopy and flow cytometry. EGFP expression was clearly observed in both the Ad Foxp3-EGFP- and Ad-EGFP-treated epithelial cells (Figure 1a). When we analyzed the single-cell suspensions from dissociated lungs after Ad Foxp3-EGFP or Ad-EGFP infection, Foxp3 was expressed in CD326^+^ epithelial cells (Figure 1d), but not in CD4^+^ or CD8^+^ cells (Figures 1b and c). These results show that i.t. administered Ad Foxp3-EGFP and Ad-EGFP were only expressed in epithelial cells.

### Foxp3 expression inhibited the influx of inflammatory cells into BALF and reduced smooth muscle cell hyperplasia in the cockroach allergen-induced asthma model

Sensitivities to cockroach allergen are significantly correlated to the rise of asthma in densely crowded inner cities with cockroach infestation.^24^

**Figure 2:**
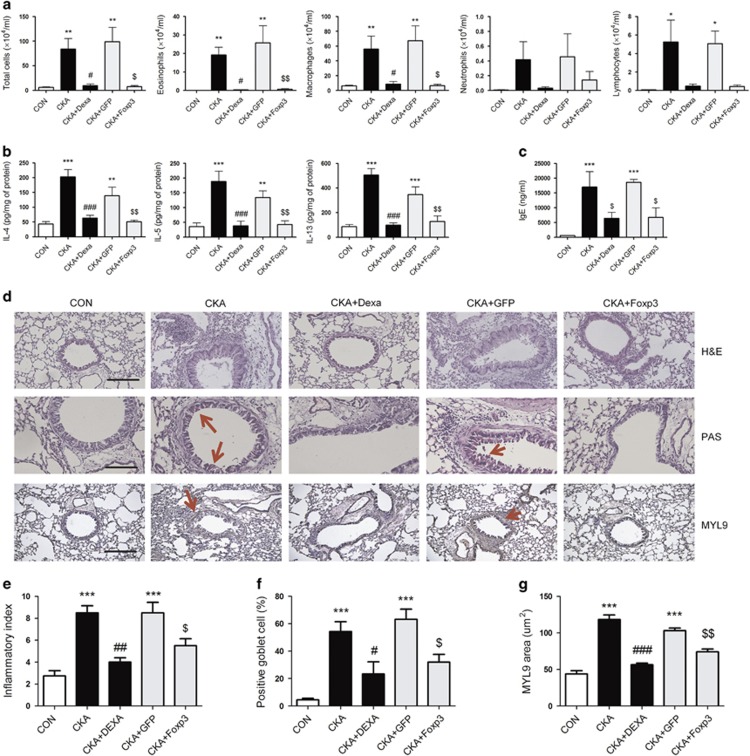
Effects of Foxp3 expression on airway inflammation in cockroach allergen-induced asthmatic mice. The total cell number, as well as the number of eosinophils, macrophages, neutrophils and lymphocytes, were counted in BALF from mouse lungs (**a**). The concentrations of IL-4, IL-5 and IL-13 in the BALF were measured by sandwich ELISA. Cytokine expression was analyzed in 100 μl of BALF and normalized to the amount of total protein in each sample (**b**). The levels of total IgE in serum were evaluated by sandwich ELISA (**c**). The effects of Foxp3 expression on histopathological changes in lung tissue during cockroach (CKA)-induced allergic asthma. Lung tissues were stained with hematoxylin and eosin (H&E), periodic acid-Schiff (PAS) and antibodies to myosin regulatory light polypeptide 9 (MYL9). Scale bars, 50 and 100 μm. The extent and the anatomical location of the leukocyte infiltrates were determined by examining H&E-stained tissue obtained from mice 24 h after the final allergen challenge (original magnification, × 200). The mucosal surfaces were stained by PAS reaction (original magnification, × 400). Mouse lung sections were incubated at 4 °C overnight with anti-MYL9 goat polyclonal antibodies (1:50). The slides were then incubated with avidin-biotin peroxidase complex, and the color was developed using 3,3′-diaminobenzidine tetrachloride. Lung tissues from PBS challenge, CKA challenge, Dexa treatment, Ad-EGFP control treatment and Ad Foxp3-EGFP treatment. Mice were processed 24 h after the last CKA challenge. A total of 6–9 mice in each group were analyzed and representative findings are shown (**d**). The degree of inflammation was quantified using a semi-quantitative scale. All of the randomly selected histological images were scored as the mean of the inflammation index (**e**). PAS-positive mucosal goblet cells around the bronchial airway were counted and are quantified as the percentage of goblet cells, as described in the Materials and methods section (**f**). The thickness of the smooth muscle layer was calculated based on immunohistochemical images (**g**). CON, PBS-treated control group; CKA, cockroach allergen-challenged group; CKA^+^DEXA, cockroach allergen-challenged and dexamethasone-treated group; CKA+EGFP, cockroach allergen-challenged and Ad-EGFP-treated group; CKA+Foxp3, cockroach allergen-challenged and Ad-Foxp3-EGFP-treated group. The data are shown as the means±s.e.m. Statistical analyses were conducted by one-way ANOVA followed by the Tukey's Multiple Comparison test (significantly different from CON, *P<0.05, **P<0.01, ***P<0.001; significantly different from CKA, ^#^*P*<0.05, ^##^*P*<0.01, ^###^*P*<0.001, significantly different from CKA+EGFP, ^$^*P*<0.05, ^$$^*P*<0.01, ^$$$^*P*<0.001, *n*=6–9).

One of the feature of asthma is the accumulation of eosinophils, neutrophils, lymphocytes and macrophages in the lungs.^25^ Before challenge with cockroach allergen, mice were i.t. infected with Ad Foxp3-EGFP or Ad-EGFP. Following challenge, the recruitment of immune cells to the airways was measured in BALF. The total number of infiltrating cells in the BALF of cockroach allergen-immunized animals was significantly higher than that of control animals. However, Ad Foxp3-EGFP treatment decreased the total number of infiltrating cells to a similar level as that observed in the dexamethasone-treated group (Figure 2a). Eosinophil, macrophage and lymphocyte numbers in the Ad Foxp3-EGFP-treated group were significantly decreased compared with those in the Ad-EGFP-treated group.

The production of Th2 cytokines such as IL-4, IL-5 and IL-13 in the BALF was considerably increased in the cockroach allergen-challenged group. In addition, cytokine levels were reduced in the dexamethasone-treated group and were comparable to those observed in the Ad Foxp3-EGFP-treated group (Figure 2b). Cross-linking of allergen-specific IgE antibodies on the surface of mast cells causes them to degranulate, subsequently leading to AHR.^26^ As elevated IgE production is a key characteristic of systemic asthmatic responses, we investigated the systemic effects of Ad Foxp3-EGFP treatment by measuring serum IgE levels. Serum IgE were significantly decreased in Ad Foxp3-EGFP-treated mice compared with that in the Ad-EGFP-treated mice (Figure 2c).

H&E staining of lung sections revealed that a greater number of eosinophils had infiltrated into the trachea and blood vessels in the cockroach allergen-challenged mice compared with control mice (Figure 2d). In addition, in asthmatic mice treated with Ad Foxp3-EGFP, eosinophil infiltration in the lung was significantly reduced. PAS staining further revealed goblet cell hyperplasia in the airways of the asthmatic mice. However, Ad Foxp3-EGFP and dexamethasone treatment both suppressed this goblet cell hyperplasia (Figures 2d–f). To evaluate airway remodeling, smooth muscle cell hyperplasia was detected by IHC using an anti-MYL9 antibody. Specifically, Ad Foxp3-EGFP treatment decreased the thickness of the smooth muscle layer compared with Ad-EGFP treatment (Figures 2d and g). These data demonstrate that adenovirus-delivered Foxp3 suppresses airway remodeling by inhibiting the recruitment of inflammatory cells.

### Foxp3 expression inhibited the influx of inflammatory cells into the BALF and suppressed airway remodeling in the OVA-induced asthma model

**Figure 3:**
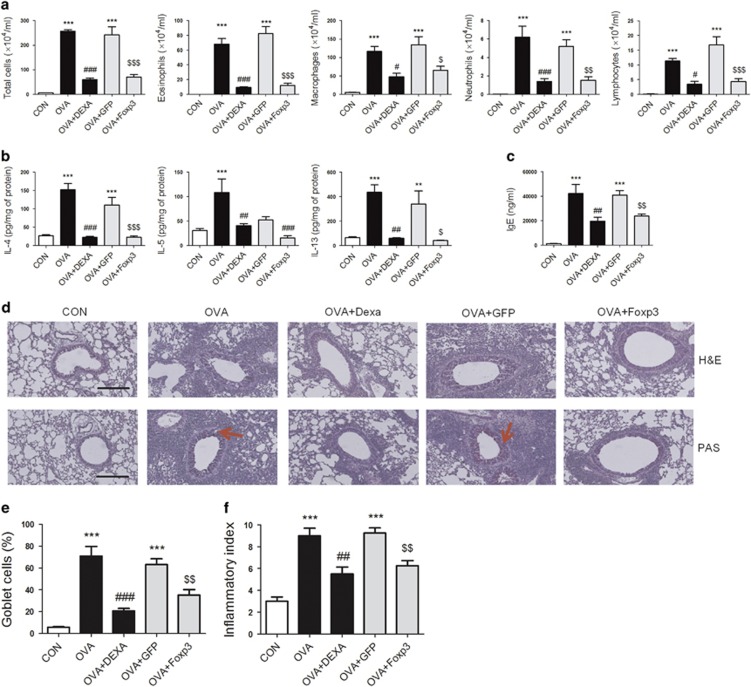
Role of Foxp3 expression on airway inflammation in OVA-induced asthmatic mice. Asthma was induced by intraperitoneal (i.p.) injection of OVA together with 20 mg of aluminum hydroxide on days 0 and 14. The mice were intratracheally (i.t.) challenged with 1% OVA in 50 μl of PBS six times between days 22 and 31. To assess the effect of Foxp3 expression on airway inflammation, mice received an i.t. instillation of Ad Foxp3-EGFP (5 × 10^8^ plaque-forming units (pfu)/mouse) on days 21 and 28. Control mice received the same dose of control virus (Ad-EGFP) or PBS. On day 32, mice were killed, blood was collected, BAL was performed, and lungs were removed for histological analysis. The number of total cells, eosinophils, neutrophils, macrophages and lymphocytes in the BALF were counted (**a**). Effect of Ad Foxp3-EGFP treatment on cytokine production in BALF. The concentrations of IL-4, IL-5 and IL-13 were measured using a sandwich ELISA kit. Cytokine expression was analyzed in 100 μl of BALF and normalized to the total protein amount in each sample (**b**). Effect of Ad Foxp3-EGFP treatment on total IgE in the serum of asthmatic mice. Blood samples were collected by cardiac puncture, and the levels of IgE in the samples were evaluated by ELISA (**c**). Mice were processed 24 h after the last OVA challenge. Tissue inflammation and goblet cell metaplasia analyzed in H&E—(original magnification: × 200) and PAS—(original magnification: × 400) stained lung sections. A total of 6–9 mice in each group were analyzed and representative findings are shown (**d**). The degree of inflammation was quantified using a semi-quantitative scale (**e**). PAS-positive mucosal goblet cells around the bronchial airways were counted and are depicted as the percentage of goblet cells (**f**). CON, PBS-treated control group; OVA, OVA-challenged group; OVA+DEXA, OVA-challenged and dexamethasone-treated group; OVA+EGFP, OVA-challenged and Ad-EGFP-treated group; OVA+Foxp3, OVA-challenged and Ad Foxp3-EGFP-treated group. The data are shown as the means±s.e.m. Statistical analyses were conducted by one-way ANOVA followed by the Tukey's Multiple Comparison test (significantly different from CON, *P<0.05, **P<0.01, ***P<0.001; significantly different from OVA, ^*#*^*P*<0.05, ^*##*^*P*<0.01, ^*###*^*P*<0.001, significantly different from OVA +EGFP, ^$^*P*<0.05, ^$$^*P*<0.01, ^$$$^*P*<0.001, *n*=6–9).

In parallel with the CKA-induced asthma model, we also evaluated the role of Foxp3 expression in models of OVA-induced asthma. OVA-mediated increases in the influx of macrophages, eosinophils, neutrophils and lymphocytes were remarkably reduced in the BALF of Ad Foxp3-EGFP-treated group compared with that of the Ad-EGFP-treated group (Figure 3a). We also evaluated serum IgE production, as well as levels of the Th2 inflammatory cytokines IL-4, IL-5 and IL-13, in the BALF (Figures 3b and c). Specifically, the levels of inflammatory cytokines and serum IgE were similar in Ad Foxp3-EGFP-infected and dexamethasone-treated (positive control) mice. However, no effects on serum IgE were observed in Ad-EGFP-infected mice.

To analyze the effects of Ad Foxp3-EGFP on histological features of asthma, H&E and PAS staining were performed on lung tissue sections. Ad Foxp3-EGFP treatment reduced the infiltration of eosinophils into the peribronchial regions of the lung. In addition, PAS-positive mucus-producing goblet cells around the airways were observed in the OVA-treated group. However, Ad Foxp3-EGFP treatment reduced the number of PAS-positive goblet cells in the airways (Figures 3d and e). To quantify these changes, blinded investigators scored H&E-stained slides using an ‘inflammatory index,' and the results showed that significant decreases in immune cell infiltration were observed in Ad Foxp3-EGFP-treated lung (Figure 3f).

### Anti-asthmatic effects of Ad Foxp3-EGFP were independent of Treg cells

**Figure 4:**
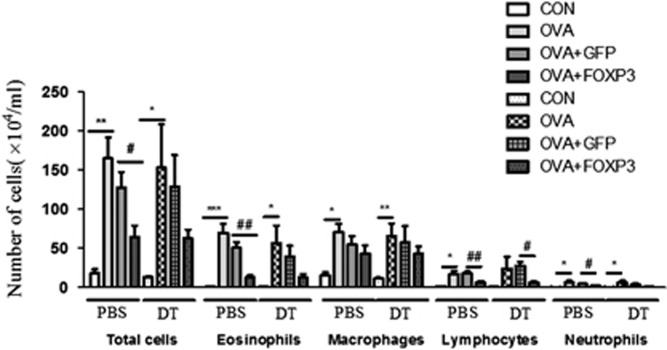
The anti-asthmatic functions of Foxp3 were independent of Tregs in OVA-induced Foxp3^*EGFP/DTR*^mice. To deplete Tregs, Foxp3^DTR^ mice were administered 0.4 μl kg^−1^ DT by i.p. injection 2 days before Ad Foxp3-EGFP or Ad-EGFP infection. The number of total cells, eosinophils, neutrophils, macrophages and lymphocytes in the BALF were counted. CON, PBS-treated control group; OVA, OVA-challenged group; OVA+EGFP, OVA-challenged and Ad-EGFP-treated group; OVA+Foxp3, OVA-challenged and Ad-Foxp3-treated group. The data are shown as the means±s.e.m. Statistical analyses were conducted by one-way ANOVA followed by the Tukey's Multiple Comparison test (significantly different from CON, *P<0.05, **P<0.01, ***P<0.001; significantly different from OVA +EGFP, ^#^*P*<0.05, ^*##*^*P*<0.01, *n*=6–9).

To examine whether the effects of Foxp3 expression on OVA-induced asthma were mediated by Treg cells, we depleted Tregs in this model by treating OVA-exposed Foxp3^DTR^ mice with diphtheria toxin. To deplete Tregs, Foxp3^DTR^ mice were administered 0.4 μl kg^−1^ diphtheria toxin (DT, Sigma-Aldrich, D0564) by intraperitoneal (i.p) injection 2 days before Ad Foxp3-EGFP or Ad-EGFP treatment. We had previously confirmed that Treg cells in the spleen are depleted by 98% after DT injection (data not shown). We then compared the recruitment of immune cells to the airways by examining BALF from these mice. Treatment of wild-type mice with Ad-Foxp3-EGFP reduced the numbers of lymphocytes and neutrophils, and these numbers were unchanged in the Treg-depleted mice. As shown in Figure 4, there were few differences in immune cell recruitment between the control (PBS) and Treg-depleted (DT) groups. Therefore, the anti-asthmatic effects of Ad Foxp3-EGFP on OVA-induced asthma were not mediated by Treg cells.

## Discussion

The airway epithelium is both a physical barrier and an essential mediator of inflammatory, immune and regenerative responses to allergens, viruses and environmental pollutants that underlie asthma pathogenesis.^27^ Airway epithelial cells recognize inhaled allergens and activate DCs to initiate various immune responses. Airway epithelial cells release CC chemokine ligand 2 (CCL2) and CCL20 in response to allergens, which facilitates the recruitment of monocytes and immature DCs to the lung.^14^ Allergen-mediated stimulation of ECs also increases the production of thymic stromal lymphopoietin, granulocyte-macrophage colony-stimulating factor (GM-CSF), IL-25 and IL-33. These cytokines activate lung DCs and promote the induction of Th2 responses.^28^

Zhang *et al.*^29^ also showed that retrovirus-mediated Foxp3 expression in lung attenuates airway inflammation in a mouse model of asthma. Specifically, they reported that enhanced Foxp3 expression reduced airway hyper-responsiveness, inflammatory cell infiltration, mucus production, and IL-4, IL-13 and IL-17 levels in this model. Although they demonstrated that Foxp3 was, indeed, expressed in murine lung tissue, they did not reveal the precise cell types in which Foxp3 was expressed (that is, epithelial cells or immune cells). Moreover, Mays *et al.* showed that overexpression of Foxp3 in mouse lung with a modified mRNA could prevent allergic asthma *in vivo.* They also showed that this modified mRNA induced Foxp3 upregulation in CD4^+^ cells.^30^ Although we used a different method of Foxp3 gene delivery (that is, adenovirus), our results are consistent with those of this previous study. The main factor that distinguishes our study from previous research is the cell type or organ site of Foxp3 gene expression. Using Foxp3-EGFP adenovirus, we detected EGFP expression only in mouse lung epithelial cells, and not in CD4^+^ or CD8^+^ cells. Furthermore, when we analyzed BALF from Treg-depleted mice, differences in the frequency of immune cells were not observed between the control and Treg-depleted groups. Therefore, in this study, adenovirus-delivered Foxp3 was predominantly expressed in lung epithelial cells.

Although we did not show the precise mechanism of Foxp3 action in lung epithelial cells, it is reasonable to presume involvement of the NF-κB pathway. Previously, it has been shown that Foxp3 could inhibit the nuclear translocation of NF-κB in T cells by increasing the stability of the NF-κB inhibitor, IκB-α.^18^ In addition, Foxp3 could also repress the transcriptional activity of NF-κB in microglia.^7^ To this end, we analyzed the effects of Foxp3 on the translocation of NF-κB in the LA-4 mouse lung epithelial cell line. Whereas NF-κB was translocated into the nucleus in EGFP control virus-infected cells after LPS stimulation, translocation of NF-κB into the nucleus was repressed in Foxp3-EGFP infected cells (unpublished data).

In conclusion, we showed that adenovirus-delivered Foxp3 was expressed in lung epithelial cells and exerted protective effects against allergen-induced leukocyte infiltration, release of pro-inflammatory cytokines and goblet cells metaplasia in asthma models. We also revealed that these effects were independent from Treg cells by using a Treg depletion model. Consistent with data from other researchers, our results have demonstrated that ectopic expression of Foxp3 could have beneficial effects for individuals with asthma.
